# Brain developmental trajectories in offspring of parents with schizophrenia or bipolar disorder

**DOI:** 10.1192/j.eurpsy.2024.47

**Published:** 2024-08-27

**Authors:** N. Van Haren

**Affiliations:** Child and adolesscent psychiatry/psychology, Erasmus Medical Center, Rotterdam, Netherlands

## Abstract

**Abstract:**

Early diagnosis and intervention are essential for managing and improving long-term outcomes of severe mental illness, highlighting the need for reliable early biomarkers. This longitudinal study explores whether the development of the brain during childhood and adolescence differs between offspring of parents with and without schizophrenia or bipolar disorder. Moreover, we will assess if the age-dependent change over time in brain volume, cortical thickness and surface, structural network indices, and cortical gyrification are related to the presence and severity of psychiatric symptoms and level of IQ.

We obtained 286 T1-weighted MRI scans of 184 offspring (aged 8–18 years at baseline) of at least one parent diagnosed with bipolar disorder (n=78) or schizophrenia (n=52) and offspring of parents without severe mental illness (n=54); 102 offspring underwent a follow-up scan (on average 3.9 years between scans).

Group comparisons and the associations with clinical and cognitive measures were analysed with linear mixed-effects models. To correct for multiple comparisons, we applied a Benjamini-Hochberg false discovery rate (FDR) correction (q=0.05).

A significant effect of age was found on most of the included brain features, with suggestive evidence for subtle deviations in trajectories in the cortical thickness, structural network indices but not in gyrification index, sulcal depth, length and width or surface area in offspring of parents with schizophrenia. Interestingly, these deviations in brain development in schizophrenia offspring remained significant after taking the presence of a diagnosis or level of IQ into account. These findings suggest the aberrant brain development in familial high-risk youngsters is associated with being at familial risk and not with (also) being at clinical high-risk.

**Disclosure of Interest:**

None Declared

